# Immunization of *Chlamydia pneumoniae* (*Cpn*)-Infected Apob^tm2Sgy^Ldlr^tm1Her^/J Mice with a Combined Peptide of *Cpn* Significantly Reduces Atherosclerotic Lesion Development

**DOI:** 10.1371/journal.pone.0081056

**Published:** 2013-12-13

**Authors:** Min Xia, Daxin Chen, Valeria Endresz, Ildiko Faludi, Andrea Szabo, Eva Gonczol, Vijay Kakkar, Xinjie Lu

**Affiliations:** 1 The Mary and Garry Weston Molecular Immunology Laboratory, Thrombosis Research Institute, London, United Kingdom; 2 MRC Centre for Transplantation, King's College London, London, United Kingdom; 3 Department of Medical Microbiology and Immunobiology, University of Szeged, Szeged, Hungary; 4 Institute of Surgical Research, University of Szeged, Szeged, Hungary; 5 Virology, National Center for Epidemiology, Budapest, Hungary; 6 Thrombosis Research Institute, Bangalore, India; King's College London, University of London, United Kingdom

## Abstract

**Objective:**

To investigate the antigenic effect of a peptide containing two epitopes of *Chlamydia pneumoniae* (*Cpn*) on atherosclerotic lesion formation in mice infected with *Cpn*.

**Materials and Methods:**

Six-week-old Apob^tm2Sgy^Ldlr^tm1Her^/J mice were immunized using a repetitive immunization multiple-sites strategy with KLH-conjugated peptides derived from the major outer membrane protein and the putative outer membrane protein 5 of *Cpn*. Mice were fed a high-fat diet and infected with *Cpn* twice during the 10-week diet period. Lesions were evaluated histologically; local and systemic immune responses were analyzed by immunohistochemistry of aorta samples and cytokine measurements in plasma samples and splenocyte supernatants.

**Results:**

Mice immunized with the combined *Cpn* peptide showed a greater reduction in lesion size compared to mice immunized with either epitope alone [54.7% vs 39.8% or 41.72%] and was also associated with a significant decrease in lesion area in descending aortas compared with those in controls (88.9% for combined Cpn peptide, 81.9% for MOMP peptide and 75.7% for Omp5, respectively). This effect was associated with a shift in the cellular composition of plaques towards decreased inflammatory cell and increased regulatory T-cell content. Additionally, the effect was also connected with decreased secretion of proinflammatory cytokines and increased production of anti-inflammatory cytokines demonstrated in plasma and in supernatant on stimulated spleen cells.

**Conclusions:**

Atherosclerotic lesion formation may be promoted by *Cpn* infection in the presence of a high-fat diet, and reduced by immunization with the combined *Cpn* peptide. The combined peptide has more potential than either epitope alone in reducing atherosclerotic lesion development through Treg expansion.

## Introduction

*Chlamydia pneumoniae* (*Cpn*) [1] is an important human pathogen that causes atypical pneumonia and is associated with various chronic inflammatory diseases such as atherosclerosis, a major cause of cardiovascular disease and death in the Western world [2]–[6]. Although the epidemiological importance of *Chlamydia* infection in atherosclerosis is not well defined, the potential role of *Cpn* in coronary atherosclerosis may be related more to acceleration of the disease or to the systemic effects of persistent infection than to sudden initiation of infarction by acute infection [7]. However, the theoretical role of *Cpn* in acceleration of atherosclerosis is still controversial [8]–[10]. Although an association between *Cpn* infection and coronary atherosclerosis has been reported, the association is less clear for the effect of peptide antigen derived from *Cpn* on the formation of atherosclerotic lesion. In addition, an epitope of the major outer membrane protein (MOMP) of *Cpn* (AA 67–74: GDYVFDRI) and the putative outer membrane protein 5 (Omp5) of *Cpn* (AA 284–292: QAVANGGAI) share high homology, with two sequence locations of ApoB protein (http://web.expasy.org/sim/). ApoB protein plays a crucial role in atherosclerosis as immunization with some peptides derived from ApoB protein reduce atherosclerotic lesion in several mouse models. Indeed, this molecular mimicry (share high homology) was recently demonstrated in our laboratory in which an epitope containing both sequences of AA 67–74 (GDYVFDRI) and AA 284–292 (QAVANGGAI) has an effect on atherosclerotic lesion reduction in a protein scaffold in non-infected mice with *Chlamydophia* [11]. In this study, we investigate the effect of a linear peptide containing these two putative epitopes derived from MOMP and Omp5 of *Cpn* on atherosclerotic lesion formation in *Cpn*-infected Apob^tm2Sgy^Ldlr^tm1Her^/J mice.

## Materials and Methods

The immunizing peptides derived from MOMP (AA 67–74: GDYVFDRI, designated as MOMP peptide) and Omp5 (AA 283–291: QAVANGGAI, designated as Omp5 peptide), and a combined peptide containing the MOMP and the Omp5 peptides (designated as a combined *Cpn* peptide) coupled by a polyglycine [(Gly)5] linker, were used in this study in a Keyhole limpet hemocyanin (KLH)-conjugated form. All of the peptides used in the study, including ApoB peptide and human HSP60 (hHSP60) peptide, were synthesized by Severn biotech Ltd (Worcestershire, UK).

### Animal Experiments

The experiments were approved by the Animal Welfare Committee of the University of Szeged and conform to the Directive 2010/63/EU of the European Parliament.

Apob^tm2Sgy^Ldlr^tm1Her^/J mice (these mice produce ApoB100 only, and are LDL receptor deficiency).were used in our study in a total of 5 groups (3 sample and 2 control groups). Each group included 6 mice (5–6-week-old males; similar body weight, 32.26±2.12 g [measured at the end of the experiment]) and the experiment was repeated. Mice were immunized with KLH-conjugated peptides mixed with Alum adjuvant subcutaneously according to a repetitive immunization multiple sites strategy (RIMMS) as described earlier [11], [12]. For infection, mice were inoculated intranasally with 2×10^6^ inclusion forming units (IFU) of *Cpn* (CV-6, cardiovascular strain) [13] in 25 µL of phosphate buffered saline (PBS) at week 4 and at week 8. This dose was chosen based on survival and symptoms observed in mice after infection with different infection doses (Table S1). CV-6 strain of *Cpn* was propagated in HEp-2 cells and partially purified as described earlier [14]. The mice were sacrificed at the end of week 12 (a high-fat diet was started at week 2 and continued for 10 weeks). For detection of *Cpn*-specific DNA, polymerase chain reaction (PCR) was performed as described by Tong and Sillis [15]. The MOMP of *Cpn* was chosen as a target for amplification in a nested PCR. All primers were synthesized in Life Technologies Ltd (Paisley, UK). The external primers (Table S2) amplified a 333 base-pair product (first-stage PCR) from the genomic DNA purified from lung homogenates of tested mice in both infected and non-infected (negative control) groups. The internal primers amplified a 207 base-pair product (second-stage PCR) using the first-stage PCR product as a template. To confirm if the mice were infected, further detection of *Cpn*-specific IgG in the mouse sera by indirect immunofluorescence was carried out. A HEp-2 cell (ATCC) monolayer grown on 13-mm coverslips in 24-well plates was infected with *Cpn* (CWL029) at a multiplicity of infection (m.o.i.) of 1. At 48 h of infection, cells were fixed with ice-cold acetone (10 min, −20°C). PBS-rehydrated cells were stained with *Cpn* MOMP-specific mouse monoclonal antibody (DAKO, Budapest, Hungary) and FITC-labelled anti-mouse IgG secondary antibody (SIGMA, Dorset, UK). Coverslips were examined under an ultraviolet-microscope, and evenly distributed bright fluorescing inclusions were visible. Mouse sera diluted in PBS were used as the primary antibody for staining the similarly treated monolayers and the same FITC-conjugated secondary antibody was applied to detect serum IgG binding to inclusions. Reciprocal of serum dilution producing clearly discernable inclusions was determined as *Cpn*-specific titre of the mouse serum. The repetitive immunization multiple-sites strategy (RIMMS) was adopted [9], [10] and mice were sacrificed at the end of week 12 (a high-fat diet was started at week 2 and continued for 10 weeks).

### Serum Lipoprotein Analysis

Serum total cholesterol, triacylglycerol and high-density lipoprotein (HDL) were measured with a Modular P800 assay system (Roche, Mannheim, Germany) through a service from the Department of Laboratory Medicine, University of Szeged, Hungary. Low-density lipoprotein (LDL) cholesterol was calculated according to the Friedewald formula:
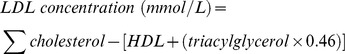



### Tissue Preparation and Antibody Response Measurements

Twelve weeks after the first immunization, tissues were harvested and mounted in Optimal Cutting Temperature (OCT) for immunohistochemical analyses and in paraffin for lesion measurement. Atherosclerosis in aortic roots was examined by Image-Pro Plus TM software, version 4.0 ((Media Cybernetics, Bethesda, MD, USA). Peptide-specific antibody titers were measured by ELISA following the manufacturer's instructions. To assess whether immunization with the combined *Cpn* peptide led to changes in lipoprotein profiles, the cholesterol, triglyceride, HDL- and LDL-cholesterol profiles of the mice were analyzed using a pooled plasma sample from each group after being fed a high-fat diet for 10 weeks.

### Immunohistochemical Analyses, Morphometric Analyses and Quantitative Measurements of Atherosclerosis

OCT-embedded samples were used for immunohistochemical analyses. Sections of paraffin-embedded tissues were stained with hematoxylin and eosin (HE) and elastin/van Gieson (Sigma) for histological examination and were evaluated using an Olympus U-ULH Optical microscope (Olympus Optical Co. Ltd, Tokyo, Japan).

Both the atherosclerotic lesions in the aortic sinus and the lesions in the descending aortas were measured. The descending aortas were evaluated for the extent of atherosclerosis.

### Measurement of Cytokines

Plasma levels of interleukin (IL)-10, transforming growth factor (TGF)-β, tumor necrosis factor (TNF)-α and interferon (IFN)-γ were measured by ELISA following the manufacturer's instructions (R&D systems, Abingdon, UK). IL-10 and TNF-α levels in the lesions were quantified by immunohistochemical analyses (rat anti-mouse TNF-α and IL-10 purchased from BioLegend, CA, USA). Levels of concanavalin-A (*Con A*)-induced IL-10, TGF-β, TNF-α and IFN-γ in splenocyte cultures were also measured.

### Fluorescence Activated Cell Sorting Analysis

Spleen cells from mice immunized with either *Cpn* peptides or KLH (control) were used for T regular (Treg) cells measurement using a Treg detection kit (Miltenyi Biotec, Surrey, UK). Cells were analyzed with a Beckman-Coulter FC-500 Analyzer (Bachman Coulter, High Wycombe, UK).

### Statistical Analyses

Data are reported as mean±standard error of the mean (±SEM), unless otherwise indicated. Figures were plotted using graph-pad Prism 5.01 and Sigma plot 9.0. For atherosclerotic lesion size, data were compared and intergroup differences were conducted using one-way ANOVA for multiple comparisons and post hoc bonferroni test. Other data were analyzed using Student's *t*-test (2-tailed analyses). Non-parametric distributions were analysized using Mann-Whitney *U* test for pairwise comparisons and the Kruskal-Wallis test for multiple comparisons. Differences between groups were considered significant at P values below 0.05.

## Results

### Detection of Cpn Infection

**Figure 1:**
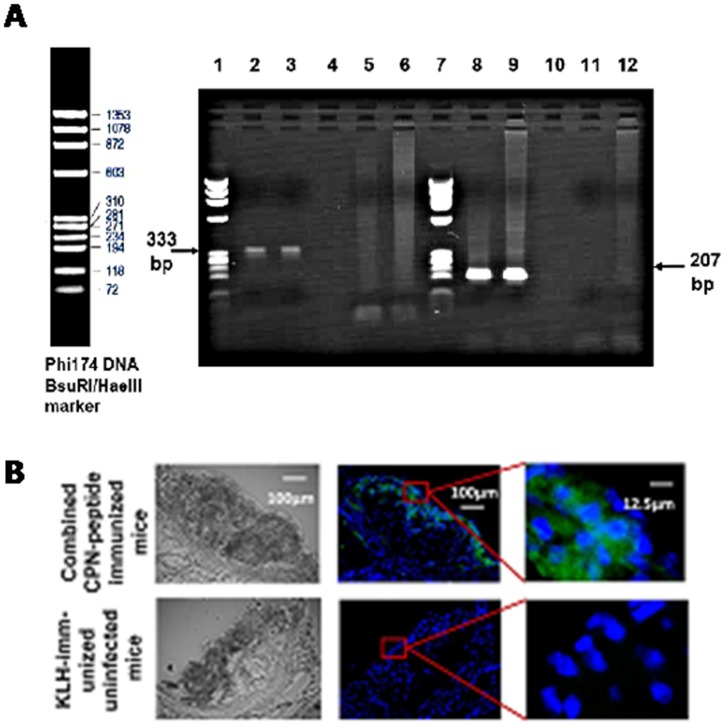
**1a.** Agarose gel electrophoresis of PCR products (first stage, lanes 2–6; second stage, lanes 8–12). Lanes 2 and 3 using genomic DNA as a template from two Apob^tm2Sgy^Ldlr^tm1Her^/J mice infected with *Cpn* bacteria; lanes 5 and 6 using genomic DNA from two non-infected Apob^tm2Sgy^Ldlr^tm1Her^/J mice; lanes 8, 9, 11 and 12 using first-stage PCR products 2, 3, 5 and 6 as a template, respectively. Lanes 1 and 7 show the Phi174 DNA/HaeIII maker (Promega). **1b.** Chlamedia LPS antibody (MCA 2718, AbD serotec) stained lesion sites in aortic sinus. *Chlamedia* LPS antibody (MCA 2718, AbD serotec) was used as the first antibody (10 mg/ml), anti-mouse IgG-FITC, developed in sheep, was used as a second antibody. Green represents *Chlamedia* LPS and blue represents cell nnucleuses stained with 4′,6-diamidino-2-phenylindole (DAPI) (Vector Lab, Peterborogh, UK) [N = 6 mice].

The MOMP of *Cpn* was chosen as a target for amplification in nested PCR DNA from infected samples only, and produced a band of the expected size after the first PCR amplification. Similarly, in the second amplification, a positive band of the correct size was obtained only from using the first PCR product as a template originally from infected and non-infected mice (Figure 1). *Cpn* DNA was detected from the lung homogenates by PCR as shown in Figure 1a, and the primers used for PCR as shown in Table S1. In addition, a genus specific epitope within *Chlamydia* lipopolysaccharde (LPS) was detected from the lesion sites of aortic sinus in *Cpn*-infected mice by *Chlamydia* LPS antibody (MCA 2718, AbD serotec) compared that of mice without *Cpn* infection (control), suggesting that *Cpn* bacteria were captured at the lesion site when mice were infected with *Cpn* bacteria as shown in Figure 1b.

### Peptide-Specific Immunoglobulin G in Sera of Immunized Mice

**Figure 2:**
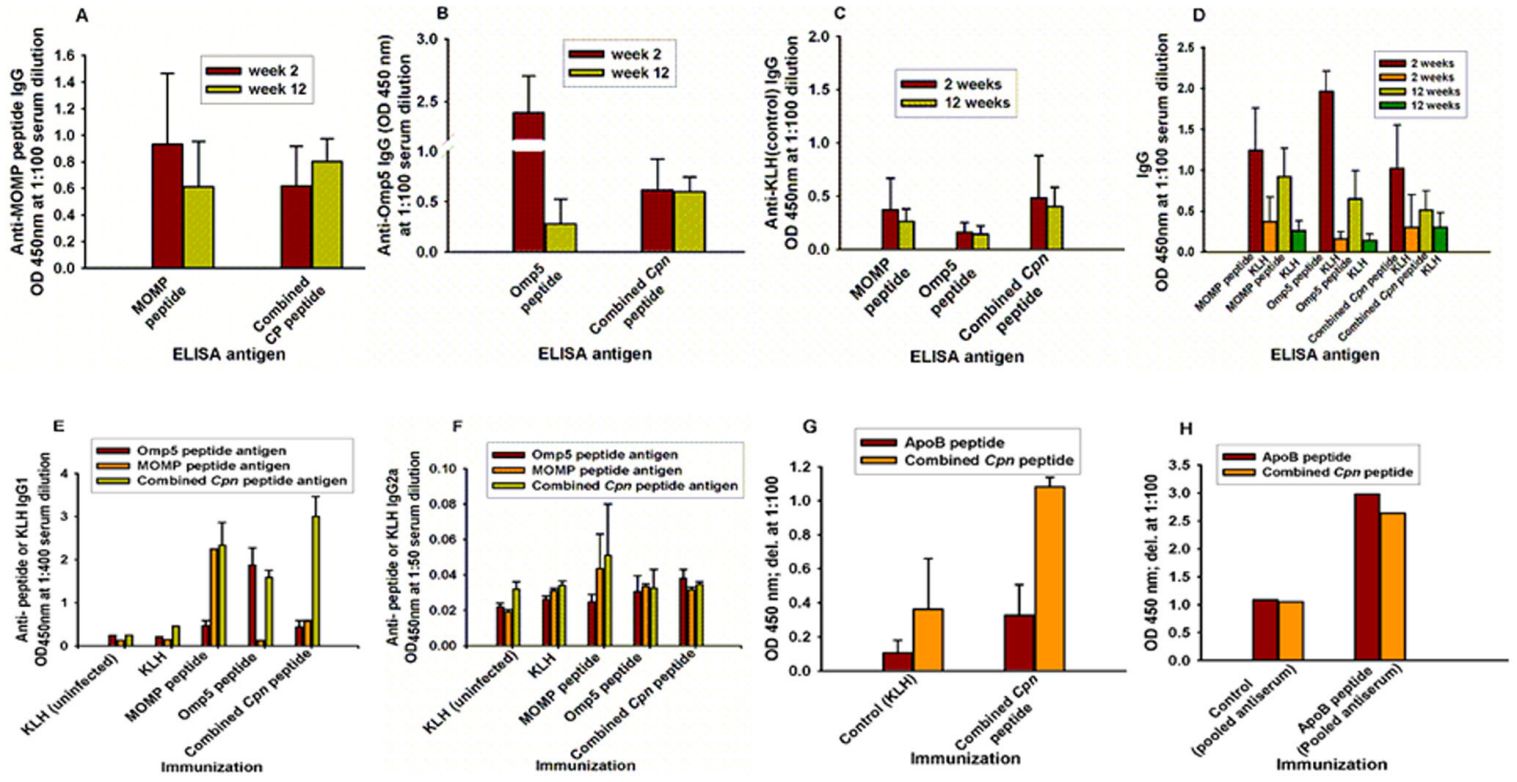
(A–C) Concentration of *Cpn* peptide-induced IgG antibodies and KLH controls in the sera of Apob^tm2Sgy^Ldlr^tm1Her^/J mice at 2 and 12 weeks after the first immunization. The mean optical densities (ODs) were obtained from plasma samples of each peptide-immunized mouse on relevant peptide-coated ELISA plates. Dilution ratio: 1∶100. (D, E) Concentrations of *Cpn* peptide-induced IgG1 and IgG2a antibodies in the sera of Apob^tm2Sgy^Ldlr^tm1Her^/J mice at 2 weeks after the first immunization. Dilution ratio: 1∶400 for IgG1 and 1∶50 for IgG2a. (F, G) Cross-reaction between the combined *Cpn* peptide and ApoB peptide (the data of Fig. 2F were from individual samples, the data of Fig. 2G were from pooled samples).

A peptide-induced specific antibody response was observed when the MOMP peptide, the Omp5 peptide and the combined *Cpn* peptide were used as ELISA antigens (Figures 2A–D), when compared with a KLH control, which produced little immune response (Figures 2C and D). The antibody immune response at week 12 was slightly lower than that at week 2 (Figure 2A–D) apart from combined *Cpn* peptide-induced antibody at week 12. High antibody concentrations against either the Omp5 peptide or the MOMP peptide at week 2 were detected in immunized mice despite a relatively lower immune response in the MOMP-peptide-immunized mice than in the Omp5-peptide-immunized mice at week 12.

### Peptide-Specific Immunoglobulin Subtypes G1 and G2a in Antiserum of Immunized Mice

A peptide-induced specific IgG1 response was observed, when compared with a KLH control (Figure 2E). There was little or no IgG2a immune response (Figure 2F) in pooled antiserum. Immunization with a KLH-conjugated ApoB and hHSP60 peptides was described earlier [11]. Sera from these experiments and sera of *Cpn* peptide-immunized mice were used for testing cross-reaction of peptide-specific antibodies. ApoB and *Cpn* peptides, individually, induced high levels of peptide-specific IgG in mouse sera two weeks after the first immunization compared to the control group immunized with KLH alone. Certain level of cross-reaction was observed between ApoB peptide and *Cpn* peptide antisera (Figure 2G), as well as between *Cpn* peptide and ApoB peptide antisera (Figure 2H) [16].

### Effect of Treatment With Cpn Peptides on Plasma Lipid Levels

Slightly lower concentrations of cholesterol and LDL were observed in the infected control mice than in the non-infected controls (Table 1). In agreement with previous report by Blessing et al, there was no significant difference observed between the infected and non-infected mice [17]. In addition, there was little change in either triglyceride or HDL concentration observed in non-infected and infected control mice as well as in mice infected after immunization with the combined *Cpn* peptide. These levels were remained the same as those in infected mice after immunization with the combined *Cpn* peptide.

**Table 1:** Plasma lipid concentrations in mice after being fed a high-fat diet for 10 weeks.

Immunization	Diet (weeks)	Cholesterol (mmol/l)	TRI* (mmol/l)	HDL* (mmol/l)	LDL* (mmol/l)
LKH-combined *Cpn* peptide-*Cpn*-infected mice	10	36.09	2.11	5.78	29.35
LKH-*Cpn*-infected mice	10	28.87	1.77	5.20	22.87
LKH-non-*Cpn*-infected mice	10	34.65	2.56	5.33	28.16

Calculated values.

### Reduction of Atherosclerotic Lesion Size in the Aortic Sinus

**Figure 3:**
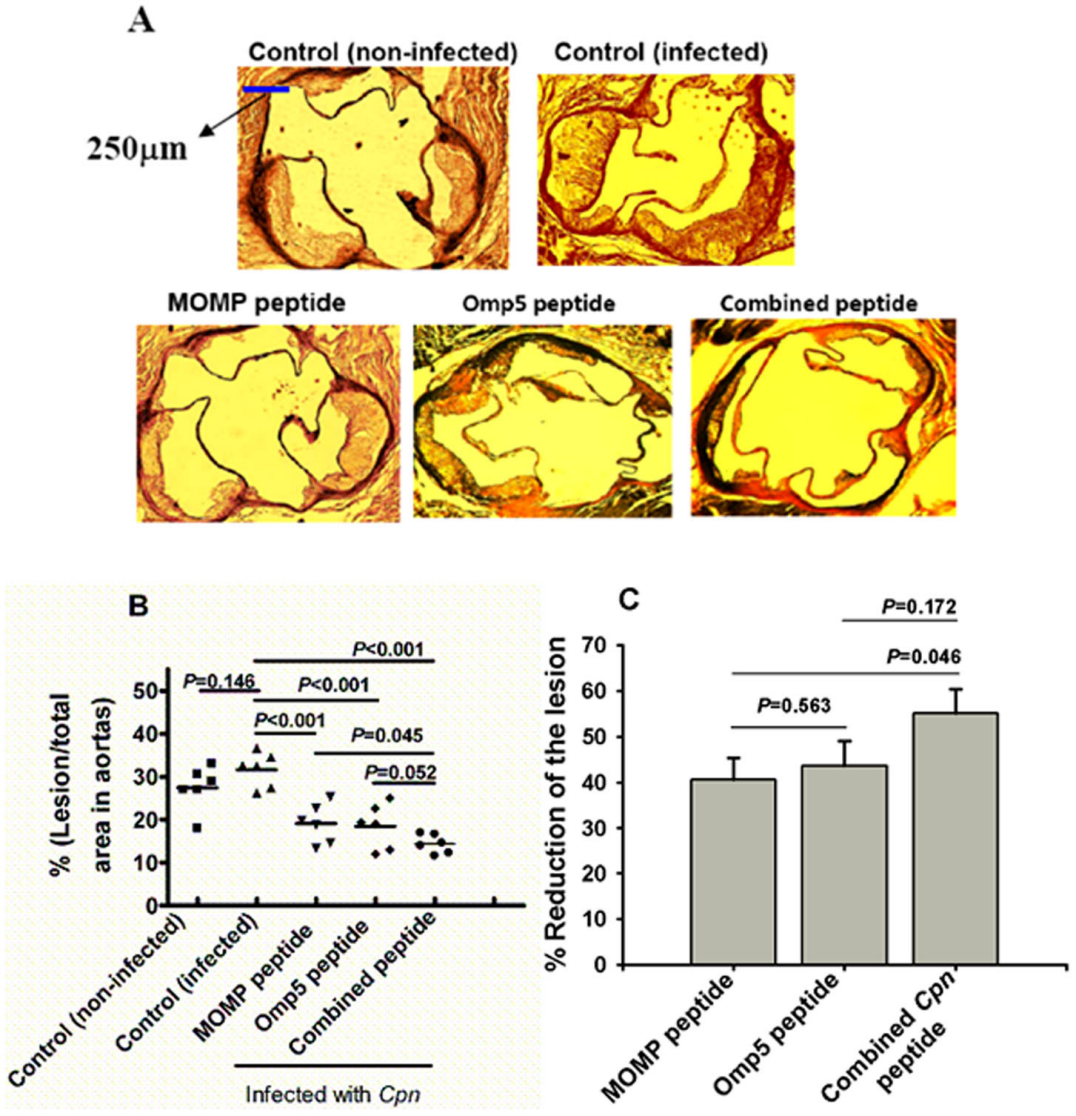
Detection and quantitation of the lesion areas in the aorta of Apob^tm2Sgy^Ldlr^tm1Her^/J mice fed a high-fat diet after immunization with each peptide antigen versus control mice immunized with KLH only (both infected and non-infected). (A) Representative photomicrographs of lesions observed in atherosclerotic aortas as analyzed with elastin/van Gieson staining. (B) Percentage of luminal surface occupied by lesions in the aortic sinus versus control mice immunized with KLH (ratio of lesion areas [µm^2^] versus total areas [µm^2^] [N = 6 mice]). (C) Reduction of lesion size shown as percentage of the lesion area versus that in control mice (infected). Data represent mean ± SEM.

Representative sections from the aortic sinuses of mice are shown in Figure 3A. The lesion size in infected control mice was 5% larger than that in non-infected controls (32% vs. 27%), but it did not show significant. In contrast, the lesion size in mice immunized with the combined *Cpn* peptide was smaller than that in either the MOMP peptide- or the Omp5-peptide–immunized mice (14% vs. 19% and 14% vs. 18%, respectively) (Figure 3B). The former pair of peptide antigens showed significant difference (*P* = 0.045), but this was not in case of the later pairs (*P* = 0.05). No significant difference in lesion size between mice immunized with either the MOMP peptide or the Omp5 peptide alone was observed.

Reduction in lesion size, expressed as a percentage of the lesion size in the aortic sinus of the control mice, was 54.7% following immunization with the combined *Cpn* peptide versus 39.8% with the MOMP peptide and 41.7% with the Omp5 peptide (Figure 3C). In addition, the lesion reduction in mice immunized with the combined *Cpn* peptide was significantly greater (*P* = 0.046) than that in MOMP peptide- immunized mice (Figure 3C).

In addition, we assessed oil red O (ORO) lipid levels in atherosclerotic lesions in the aortic sinus. Representative sections from the aortic sinuses of mice are shown in Figure S1A. The lipid level at the lesion sites in infected control mice was approximately 7-fold higher than in the MOMP-peptide-immunized mice (32.5% versus 4.6%, respectively), 6- fold higher than in the Omp5-peptide-immunized mice (32,5% versus 5.2%, respectively) and 9-fold higher than in the combined *Cpn* peptide-immunized mice (32.5% versus 3.6%, respectively) (Figure S1B). Lipid level in mice immunized with the combined peptide was significantly lower than that in either the MOMP-peptide-immunized mice (4.6% versus 3.6%, *P* = 0.028) or the Omp5-peptide-immunized mice (5.2% versus 3.6%, *P* = 0.001), respectively.

Furthermore, we examined the impact of treatment with *Cpn* peptides on the collagen in these lesions. The reduction of atherosclerosis in mice treated with these peptides was associated with an increased collagen content: 4-fold for the combined *Cpn*- peptide-immunized mice versus control mice (24.4±1.2% versus 5.9±0.5%; *P*<0.001), 3-fold for the MOMP peptide-immunized mice (19.4±1.3% versus 5.9±0.5%; *P<*0.001), and 3-fold for the Omp5 peptide-immunized mice (18.9±1.5% versus 5.9±0.5%; *P*<0.00) (Figures S2A and B), respectively. Mice immunized with the combined peptides showed a significant collagen increase compared to mice immunized with either the MOMP- or the Omp5-peptide alone (*P = *0.007 and *P = *0.004, respectively).

### Reduction of Atherosclerotic Lesion Size in Descending Aortas

**Figure 4:**
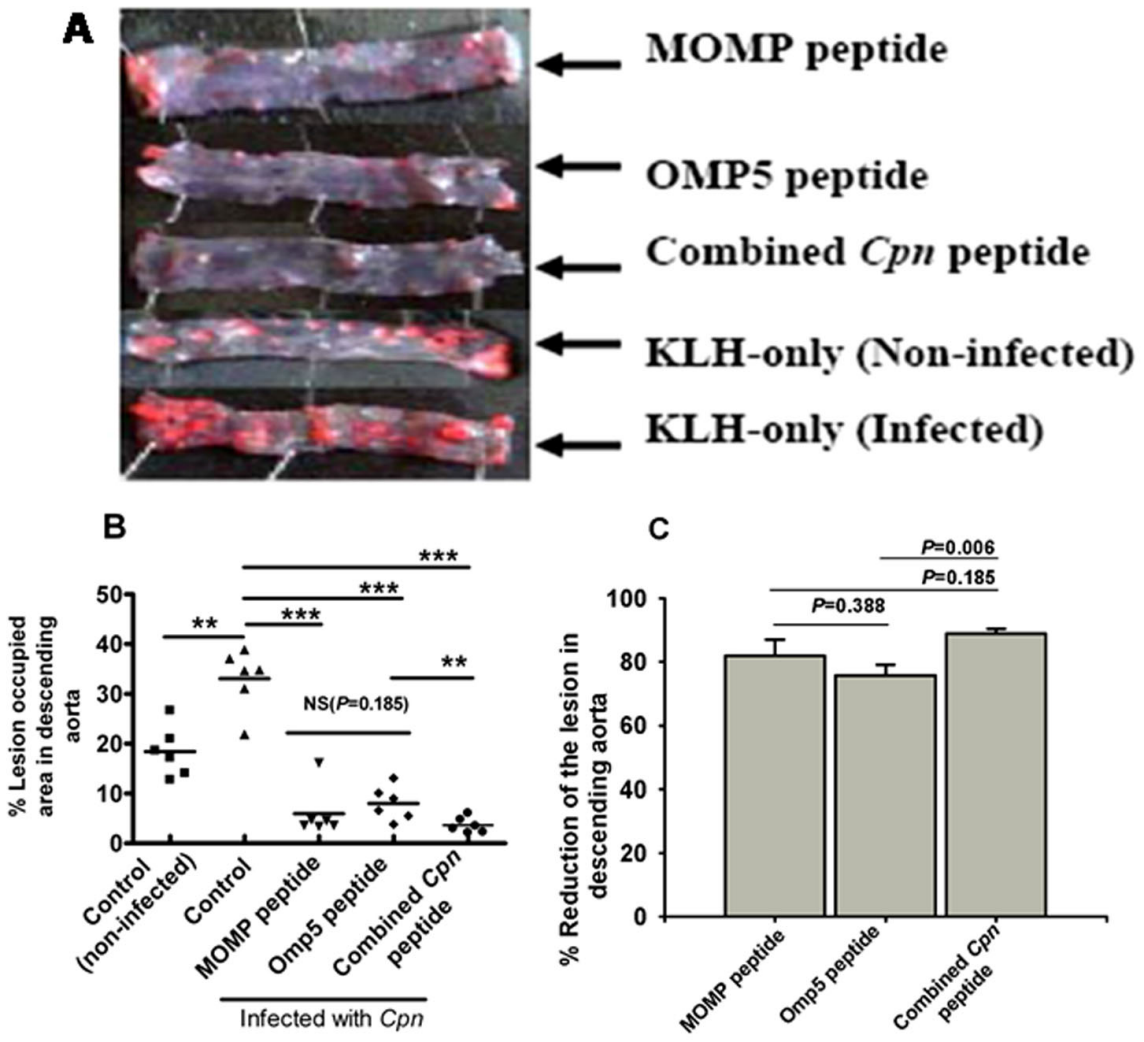
Detection and quantitation of lesion areas from *en face* descending aorta of Apob^tm2Sgy^Ldlr^tm1Her^/J mice fed a high-fat diet after immunization with each peptide antigen versus control mice immunized with KLH only. (A) Representative stained *en face* descending aorta from mice infected with *Cpn*. (B) Percentage of lesion-occupied area versus total area. (C) Percentage reduction of the lesion. Data represent mean ± SEM. *P<0.05; ** *P*<0.01; ***P<0.001.

Lesion size in the descending aortas of infected control mice was greater than that in non-infected control mice (32.0% vs. 17.0%, *P*<0.01) (Figure 4A). Lesion size in the descending aortas in mice immunized with the combined peptide was smaller than that in either the MOMP-peptide-immunized (3.4% vs. 5.8%) or the Omp5-peptide-immunized (3.4% vs. 7.8%) mice respectively (Figure 4B). There was, however, no significant difference in lesion size between mice immunized with either the MOMP peptide or the Omp5 peptide, or between mice immunized with either the MOMP peptide or the combined *Cpn* peptide; a significant difference (*P*<0.01) was found between mice immunized with the Omp5 peptide versus the combined *Cpn* peptide. Reduction in lesion size, expressed as a percentage of lesion size in descending aortas, was 88.9%, 81.9% and 75.7% from mice immunized with the combined *Cpn* peptide, the MOMP peptide and the Omp5 peptide, respectively, showing a similar trend to that observed in aorta sinus (Figure 4C). The calculated percentage reduction in lesion size showed a significant difference (*P* = 0.006) between mice immunized with the Omp5 peptide versus the combined *Cpn* peptide (Figure 4C).

### Amount of Inflammatory Cells in The Atherosclerotic Lesions

**Figure 5:**
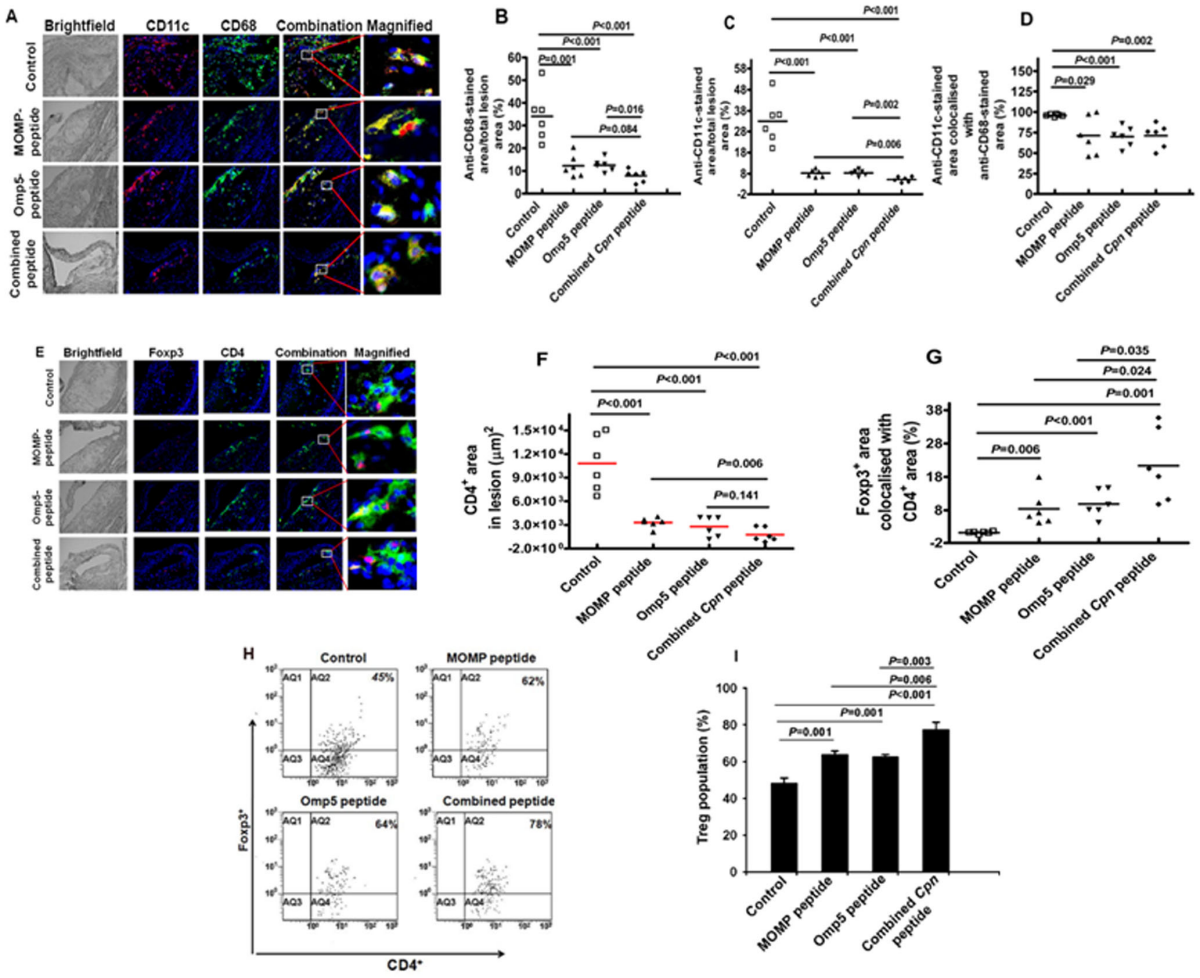
(A–D) Assessment of inflammation-associated cells in the lesions of Apob^tm2Sgy^Ldlr^tm1Her^/J mice fed a high-fat diet after immunization with peptide antigens. (A) Representative photomicrographs showing immunohistochemical staining of CD68 (green) and CD11c (red) markers, respectively. Percent occupied lesion (vs. infected control) for (B) CD68 and (C) CD11c. Magnification: 400×. Data represent the mean value ± SEM. (D) Assessment of inflammation-associated cells as percentage of CD11c areas co-localized with CD68 area (N = 6 mice). (E–G) Assessment of CD4^+^ Treg cells in the lesions of Apob^tm2Sgy^Ldlr^tm1Her^/J mice fed on a high-fat diet after immunization with peptide antigens. (E) Representative photomicrographs showing immunohistochemical staining of CD4^+^ (green) and Foxp3^+^ Treg (red) cells. Magnification 800×. (F) Observation of CD4^+^ occupied lesion area (N = 6 mice). (G) Assessment of Treg cells as percentage of Foxp3^+^ areas co-localized with CD4^+^ area (N = 6 mice). (H) Representative flow cytometric plots for CD4^+^CD25^+^Foxp3^+^ (Treg) cell population in spleen cells. Spleen cells from mice immunized with either *Cpn* peptides or KLH (control) were performed using a Treg detection kit (Ailtenyi Biotec, Surrey, UK) according to manufacturer's protocols. (I) Bar chart presentation of flow cytometric analysis. Data represent mean±SEM of data from 3 independent samples.

The percentage of macrophage-occupied area in the lesions in mice immunized with the combined peptide was 7.8%, significantly lower than that in mice immunized with the Omp5 peptide (12.2%, *P* = 0.016), but not with the MOMP peptide (12.3%, *P* = 0.084) alone. In contrast, mice immunized with the KLH showed a 34.1% occupation (Figures 5A and B).

The proportion of anti-CD11c^+^-stained lesion area was 5.2±0.4% in mice immunized with the combined *Cpn* peptide, lower than that with either the MOMP peptide (8.3±0.5%, *P = *0.006) or the Omp5 peptide (8.4±0.5%, *P* = 0.002) alone, and the control group (32.9±2.8%, *P*<0.001) (Figure 5C). In addition, the proportion of anti-CD11c^+^-stained lesion area co-localized with CD68^+^ was 71.2±4.5% for the combined peptide, 77.7±9.5% for the MOMP peptide and 74.6±7.0% for the Omp5 peptide, compared with 95.7±0.8% for the controls (Figure 5D).

Forkhead box P3 (Foxp3) is a transcriptional regulator of CD4^+^CD25^+^ regulatory T cells [18]. Thus, we chose to investigate whether the compromised suppressor function of Tregs observed in mice immunized with these peptides was associated with Foxp3 up-regulation. We found that the CD4^+^-occupied area in the lesion was significantly reduced in mice immunized with these peptides (*P<*.0.001) compared with that of control mice immunized with the KLH only (3308±268 µm^2^ versus 10,607±1435 µm^2^ for the MOMP peptide, 2779±525 µm^2^ versus 10,607±1435 µm^2^ for the Omp5 and 1761±359 µm^2^ versus 10,607±1435 µm^2^ for the combined *Cpn* peptide, respectively) (Figures 5E and F). In addition, the CD4^+^-occupied area in lesions in mice immunized with the combined *Cpn* peptide developed significantly smaller occupied areas in lesions than those of mice immunized with the MOMP peptide (P = 0.006) (Figures 5E and F). In contrast, the proportion of Foxp3^+^ area co-localized with CD4^+^ area in mice immunized with these peptides was increased compared with that in control mice immunized with the KLH alone, showing 21.4% (*P* = 0.001) for the combined *Cpn* peptide, 8.4% (*P* = 0.006) for the MOMP peptide, and 10.0% (*P*<0.001) for the Omp5 peptide, versus 1.1% for the control (Figures 5E and G). In addition, a statistically significant difference (*P*<0.05) was observed between the combined *Cpn* peptide and either the MOMP peptide or the Omp5 peptide (Figures 5E and G).

Consistently, the flow cytometric analysis of spleen cells showed significantly increased expression of Foxp3^+^ in mice immunized with these peptides compared with that of the KLH control mice (*P*≤0.001, Figures 5H and I). Similar to the observation in the lesions, higher expression of Foxp3^+^ was found in mice immunized with the combined *Cpn* peptide than those of mice immunized with either the MOMP peptide (*P* = 0.006) or the Omp5 peptide (*P* = 0.003).

### Expression of Anti-Inflammatory Cytokines and Pro-Inflammatory Cytokines

**Figure 6:**
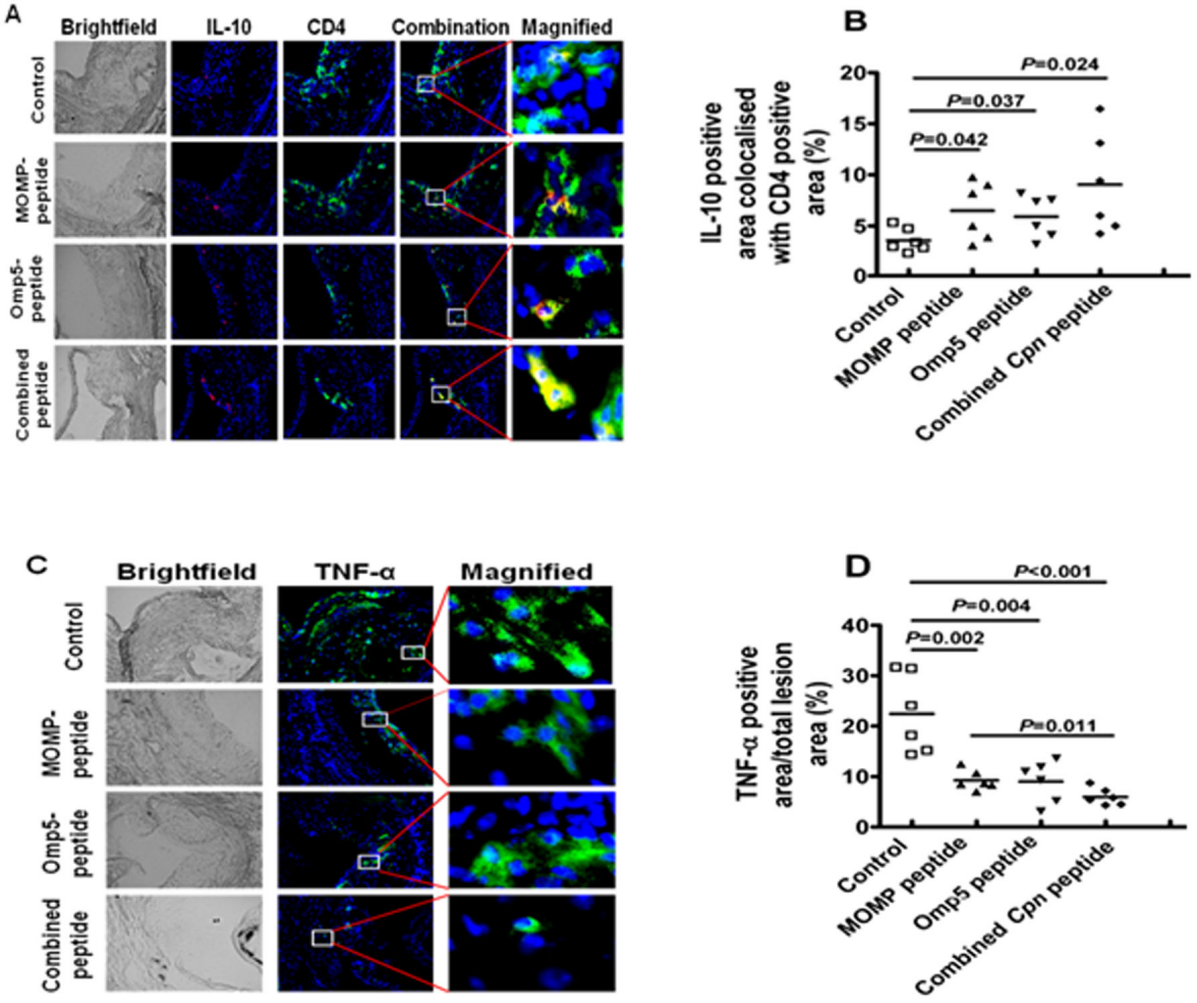
Assessment of interleukin-10-producing T cells and tumor necrosis factor-α expression in the lesions of Apob^tm2Sgy^Ldlr^tm1Her^/J mice fed a high-fat diet after immunization with peptide antigens. (A) Representative photomicrographs showing dual-immunohistochemical staining for IL-10 (red) and CD4 (green). (B) Percentage of IL-10-positive area co-localized with CD4^+^ area. (C) Relative ratio of immunohistochemical stained area (green) for TNF-α in the lesion versus total lesion area. (D) Percentage reduction of TNF-α-positive area versus that in the control mice (N = 6 mice).

To further characterize the effect of immunization with the combined *Cpn* peptide, we analyzed IL-10 expression in aortic lesions of mice immunized with these peptides (Figure 6A). The proportion of CD4^+^ cells expressing IL-10 was significantly higher in mice immunized with the combined *Cpn* peptide compared with control mice (>2-fold; 9.0% versus 3.6%, *P* = 0.024). Mice immunized with either the MOMP peptide or the Omp5 peptide showed similar proportions of IL-10 (6.0% and 5.6%, respectively) and showed significantly higher proportions in lesion sites compared with that in controls (*P*<0.05). Mice immunized with the combined *Cpn* peptide had a greater IL-10-positive area compared with either the MOMP peptide or the Omp5 peptide alone; but this increased level did not show statistical significance (Figure 6B).

Immunohistochemical analyses of TNF-α showed significantly smaller TNF-α-occupied areas in lesions of mice immunized with the combined *Cpn* peptide compared with controls (6.0% for the combined peptide vs. 9.2% for the MOMP peptide, 9.0% for the Omp5 peptide and 22.4% for the controls). The difference was statistically significant for the MOMP peptide (*P*<0.05), but not for the Omp5 peptide when compared with the combined *Cpn* peptide (Figures 6C and D).

### Level of Atheroprotective and Atherogenic Cytokines in Plasma and in Supernatants of Stimulated Splenocytes

**Figure 7:**
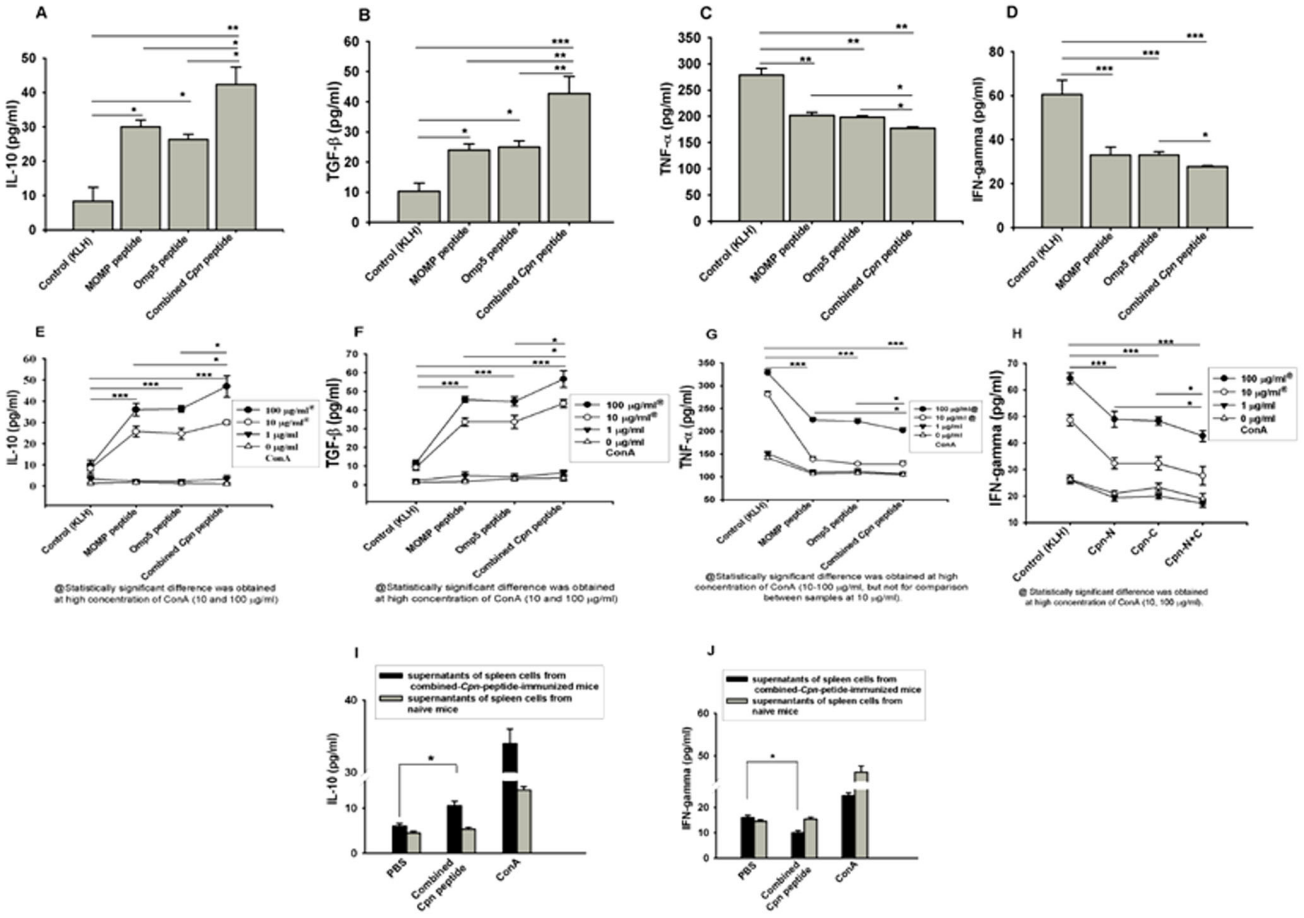
(A–D) Plasma concentrations of cytokines in Apob^tm2Sgy^Ldlr^tm1Her^/J mice versus controls after immunization with peptide antigens. (E–H) Concentrations of cytokines in the supernatant of splenocytes stimulated with Apob^tm2Sgy^Ldlr^tm1Her^/J Mice fed with a high-fat diet after immunization with peptide antigens versus infected controls (N = 6 mice). *P<0.05; **P<0.01; ***P<0.001.

Plasma levels of atheroprotective cytokine IL-10 (*P*<0.05) and TGF-β (*P*<0.01) were significantly increased in mice immunized with the combined *Cpn* peptide compared with either the MOMP peptide or the Omp5 peptide (Figures 7A and B). The levels of these two cytokines were also significantly increased in mice immunized with either the MOMP peptide (*P*<0.05) or the Omp5 peptide (*P*<0.05) (Figures 7A and B) versus controls. Plasma levels of TNF-α were significant reduced by immunization with the combined peptide compared with either the MOMP (*P*<0.05) or the Omp5 peptide (Figure 7C); all peptides produced a reduction in TNF-α secretion versus controls. A similar trend was obtained for these peptides in respect of plasma levels of IFN-γ (Figure 7D). Although no statistically significant difference was found between the combined *Cpn* peptide and the MOMP peptide, a significant difference in IFN-γ levels was found in mice immunized with the combined *Cpn* peptide compared with the Omp5 peptide (*P*<0.05) (Figure 7D).

In addition, supernatants of the splenocytes from mice immunized with these peptides individually showed significantly increased secretion of IL-10 (Figure 7E) and TGF-β (Figure 7F), stimulated with 10 or 100 µg/mL of Con A (*P*<0.001). The incremental secretion of IL-10 and TGF-β in the combined *Cpn* peptide-immunized mice was significantly higher than either the MOMP or the Omp5 peptide-immunized mice (*P*<0.05). Significantly decreased secretion of TNF-α (Figure 7G) and IFN-γ (Figure 7H) was found in the supernatants of splenocytes in mice immunized with these peptide antigens. A significantly greater decrease in the secretion of TNF-α was observed in mice immunized with the combined *Cpn* peptide versus immunized with either the MOMP peptide or the Omp5 peptide alone (Figure 7G; *P*<0.05) stimulated with 100 µg/mL of ConA (*P*<0.05). A significantly greater decrease in secretion of IFN-γ was detected in the combined *Cpn* peptide-immunized mice versus those in either the MOMP-peptide or the Omp5 peptide-immunized mice (Figure 7H, *P*<0.05), stimulated with 10 or 100 µg/mL of ConA (*P*<0.05).

Furthermore, IL-10 concentration was significantly higher in the supernatant of spleen cells from the combined *Cpn* peptide-immunized mice stimulated by the same antigen peptide than that after stimulation by PBS (*P*<0.05, Figure 7I). In contrast, IFN-γ was significantly lower in supernatant of spleen cells from the combined *Cpn* peptide-immunized mice stimulated by the same antigen peptide than that of mice stimulated by PBS (*P*<0.05, Figure 7J). In the similar experimental conditions, supernatant of spleen cells from the naive mice failed to show these differences.

Moreover, CD4^+^ expressing IL-17A levels in splenocytes from immunized mice with either *Cpn* peptides or KLH alone were also assessed. Representative flow cytometric plots for population of IL-17A in CD4^+^ T cells from splenocytes are shown in Figure S3A. CD4^+^ expressing IL-17A level in control mice was 3.2% larger than the MOMP peptide-immunized mice (5.0% versus 1.8%), 2.4% larger than the Omp5-peptide-immunized mice (5.0% versus 2.6%), and 3.8% larger than the combined *Cpn-*peptide-immunized mice (5.0% versus 1.2%) (Figure S3B). In addition, a significant difference was observed between the combined *Cpn*-peptide-immunized mice and either the MOMP-peptide- (*P*<0.011) or the Omp5-peptide-immunized mice (*P*<0.011) as well as between the MOMP-peptide- and the OMP5-peptide-immunized mice (*P*<0.001).

### Evaluation of Antigen-Induced Specific Treg Function and Specific Cellular Immune Response

**Figure 8:**
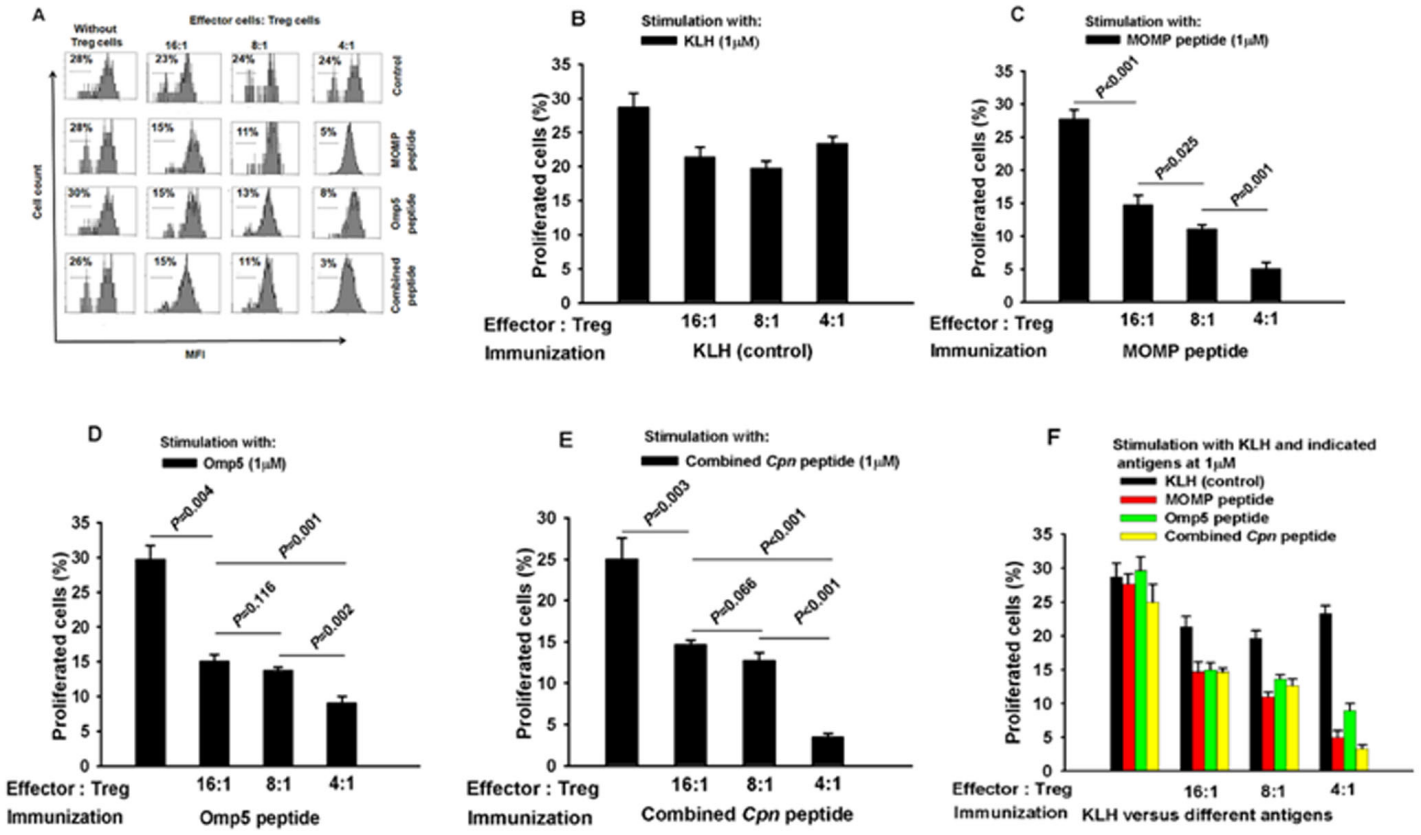
Assessment of antigen-specific regulatory function in antigen-immunized mice. Inhibition of CD4^+^CD25^−^ effector T-cell proliferation by CD4^+^CD25^+^ regulatory T–cells isolated from control (KLH–immunized) mice (A,B,F) and peptide-immunized mice when the MOMP- peptide (A,C,F), the Omp5-pepide (A,D,F), and the combined peptide (A,E,F) were used as antigens. Proliferation of effector cells isolated from immunized mice alone is indicated in the leftmost bar of each group. Addition of Treg cells to T-effector cells at different ratios was also shown. Data are expressed as mean of 6 analyses ± SEM.

To assess whether functional Treg cells were induced by immunization, we co-cultured antigen-specific Treg cells with CD4^+^ effector T-cells (CD4^+^CD25^-^ T-cells). Proliferation of effector T-cells from control mice immunized with KLH in response to stimulation with KLH at 1 µM did not show suppression in a dose-dependent manner in the presence of Treg cells from KLH-immunized mice (Figures. 8A, B and F). In contrast, Inhibition of effector T- cell proliferation was achieved from sampling mice immunized with either the MOMP peptide (Figures 8A, C and F), or the Omp5 peptide (Figures 8A, D and F) as well as the combined peptide (Figures 8A, E and F), when co-cultured CD4^+^CD25^−^ effector T cells with CD4^+^CD25^+^ Treg cells isolated from these mice in response to stimulation with related antigen. The differences were significant when adding Tregs to the effector cells at the ratios between 4∶1∼16∶1 (P<0.05∼<0.001) compared with that without the addition of Tregs. Additionally, the differences were significant when compared different concentration of added Treg in sampling mice at the ration between 4∶1∼16∶1 of Treg and T effector cells (P<0.05∼<0.001).

## Discussion

In this study, we investigated the effect of the combined peptide derived from *Cpn* proteins of MOMP and Omp5 in modulating experimental atherosclerosis in Apob^tm2Sgy^Ldlr^tm1Her^/J mouse-a strain that develops high levels of atherosclerosis after being fed a high-fat diet [16]. The effect of immunization with the combined peptide was compared with that of the two peptides used singly. The two peptides were selected for coupling based on our hypothesis that they share high homology with ApoB protein which is associated with atherosclerosis.

In line with previous reports by Blessing et al [17] and Ezzahiri et al [19], our results showed that infection of mice with *Cpn*bacteria promotes the formation of atherosclerotic lesion. Unlike that reported by Blessing et al, our results did not show statistical significance on promoting the lesion in mice infected with *Cpn* bacteria compared with that of uninfected mice following feeding with a high-fat diet for 10 weeks. These results suggest that feeding with a high-fat diet for longer than 10 weeks may be necessary to form sufficient lesion area, as the data from Blessing et al have shown that the fast lesion increasing time seems between 10–16 weeks [17]. Additionally, other factors, such as different mouse or bacterial strains and different inoculating doses may also affect lesion formation. We report the novel finding that either the MOMP peptide or the Omp5 peptide significantly reduces atherosclerotic lesion when used as a peptide antigen in Apob^tm2Sgy^Ldlr^tm1Her^/J mice infected with *Cpn* bacteria. A different study was shown that a recombinant protein containing both of the MOMP peptide and the Omp5 peptide sequence significantly reduced atherosclerotic lesion in non-infected mice [11], indicating infection of mice with *Cpn* is an independent factor for functional study of these two peptides. The mechanism of this function may be due to molecular mimicry, as the combined *Cpn* peptides can cross-react with antiserum induced by human ApoB peptide (also called ApoB100_661–681_ peptide). Although certain level of cross-reaction was observed between ApoB peptide and *Cpn* peptide antisera, strong cross-reaction (approx net 1.5 OD value) between *Cpn* peptide and ApoB peptide antisera was striking. In fact, autoantibodies to this ApoB peptide as well as to other ApoB100 peptides are present in human plasma and are associated with decreased cardiovascular risk. In addition, immunization with this peptide reduces atherosclerotic lesion in several knock-out mouse models including LDL receptor^−/−^/human ApoB-100 mice [20], ApoE^−/−^ mice [21] and Apob^tm2Sgy^Ldlr^tm1Her^/J mice [16]. Vaccination with a modified *Streptococcus pneumoniae* (*Spn*) has been reported to decrease atherosclerotic lesion formation through molecular mimicry between *Spn* and oxidized low-density lipoprotein (oxLDL) [22], which has been demonstrated to play a role in the development of atherosclerotic lesions [23], [24]. Immunization of oxLDL or apolipoprotein B (ApoB) peptide of oxLDL reduces atherosclerotic lesion formation [16], [25] in either ApoE^−/−^ or Apob^tm2Sgy^Ldlr^tm1Her^/J mouse models. However, molecular mimicry can occur in the absence of any true sequence homology between *Cpn*-derived peptide and ApoB peptide by using the computer-based software ‘SIM-Alignment Tool’ for protein or peptide sequences (http://web.expasy.org/tools/sim), in agreement with the report by Kohm et al [26]. In this case, it could explain why antigenic surfaces, rather than sequence homology, dictate molecular mimicry [27].

The data from our present study also demonstrate that reduction of the lesion, either in the aortic sinus or the descending aorta, in mice immunized with the combined *Cpn* peptide is significantly greater than with either the MOMP- or the Omp5-peptide alone. In addition, the reduction in lipid level at lesion sitesin mice immunized with the combined *Cpn* peptide is significantly greater than that in mice immunized with either the MOMP or the Omp5 peptide alone. Furthermore, the reduction of atherosclerotic lesion in mice treated with these peptides was associated with an increase in collagen content. Collagen content in mice immunized with the combined *Cpn* peptide is significantly greater than that in mice immunized with either the MOMP- or the Omp5-peptide alone. These data suggest that the *Cpn*-derived combined peptide may have an additive effect.

Immunization Keyhole limpet hemocyanin (KLH) with the combined *Cpn* peptide was associated with intracellular responses that influence cellular infiltration into atherosclerotic lesions, as the levels of macrophages, activated CD4^+^ T cells and dendritic cells (markers of early lesion formation) were decreased compared with those in controls [28]. In addition, CD4 T-cells from the mice immunized with the combined *Cpn* peptide harbor 2-fold more Foxp3^+^ Treg cells compared with either the MOMP peptide or the Omp5 peptide and approximately 20-fold more compared with controls. As Th2-type IgG1, and not Th1-type IgG2a, anti-*Cpn* peptide antibody titers were observed, indicating an association of *Cpn* peptide treatment with Th2 response which was enhanced by Treg [29], [30].

It appears that there is a shift toward decreased pro-inflammatory cytokine secretion and increased anti-inflammatory cytokine production in antigen-immunized mice, as the main biological function of IL-10 and TGF-β is to limit and terminate the inflammatory responses [31]. IL-10 has been reported to be a global suppressor of immune responses as well as an immunoregulator of the Th-2 cell response [31] while IL-17A (previously called IL-17) is a unique T helper lineage that regulates tissue inflammation [32]. Evidence was obtained from CD4^+^ expressing pro-atherogenic cytokine IL-17A [33], [34], which was shown decreased IL-17A population in peptide antigen-immunized mice in this study.

The suppressive function of CD4^+^CD25^+^Foxp3 ^+^ natural Tregs appears to require only cell–cell contact or proximity in vitro, whereas the in vivo function of these cells is associated with secretion of IL-10 and TGF-β [11], [35]. We demonstrated in vitro that the atheroprotective effect paralleled an induction of Treg suppression of antigen-specific effector T-cells, thus suggesting Tregs have an active role in the control of the atherosclerotic process. Treg cells are characterized by the expression of Foxp3, which has a crucial role in their suppression function [36].

Collectively, our study has provided evidence that the mechanism of lesion reduction by subcutaneous immunization with *Cpn*-derived hepta- or nona-peptide is duo to antigen-induced specific Treg expansion, which suppresses T effector cell proliferation along with increased atheroprotective cytokines and decreased proinflammatory cytokines. Notably, IL-17A that was thought to play a pro-atherogenic role in atherosclerosis [37], [38] was down-regulated, however, the relevance of IL-17A to human atherosclerosis remains poorly defined.

In conclusion, we have provided proof of principle evidence that the combined *Cpn* peptide antigen has an additive effect as this peptide in most of experiments, if not all, showed statistically significant difference when compared to either the MOMP- or the Omp5- peptide antigen used singly (Table S3). It appears that modulation of atherosclerosis-related autoimmunity by antigen-specific activation of Tregs represents a novel approach for the development of bivalent vaccines against atherosclerosis.

## Supporting Information

Figure S1 Detection and quantitation of lesion areas in the aorta of *Ldlr^tm1Her^Apob^tm2Sgy^*J mice infected with *Cpn* bacteria and fed a high-fat diet after immunization with *Cpn* peptides vs controls infected with *Cpn* bacteria and fed a high-fat diet after immunization with KLH only. **A.** Representative photomicrographs of Oil Red O staining for lipids in cryosections of aortic root from immunized mice. Lipids are identified by red color. **B.** Quantification of ORO staining in the aortic root of Apob*^tm2Sgy^*Ldlr*^tm1Her^*/J mice. ORO stained area versus total area (%) at aortic roots (N = 18 sections, 3 sections per mouse).
(TIF)

Figure S2 Detection and quantitation of collagen contents at lesion areas in the aorta of *Ldlr^tm1Her^Apob^tm2Sgy^*J mice infected with *Cpn* bacteria and fed a high-fat diet after immunization with *Cpn* peptides vs controls infected with *Cpn* bacteria and fed a high-fat diet after immunization with KLH only. **A**, Representative photomicrographs and quantitative analysis of collagen (Sirius Red coloration under polarized light) in atherosclerotic aortas in individual mice **B**. Quantitation of collagen content at lesion area in the aorta of Apob*^tm2Sgy^*Ldlr*^tm1Her^*/J mice (N = 18 sections, 3 sections per mouse).
(TIF)

Figure S3 Assessment of IL-17A expression level in splenocytes from *Ldlr^tm1Her^Apob^tm2Sgy^*J mice infected with *Cpn* bacteria and fed a high-fat diet after immunization with *Cpn* peptides vs controls infected with *Cpn* bacteria and fed a high-fat diet after immunization with KLH only. **A.** Representative flow cytometry plots for IL-17A expressing CD4+ population in spleen cells. Spleen cells from mice infected with *Cpn* bacteria and fed a high-fat diet after immunization with *Cpn* peptides and control mice infected with *Cpn* bacteria and fed a high-fat diet after immunization with KLH only were purified using a CD4^+^ purification kit (Miltenyi Biotec, Surrey, UK) according to manufacturer's protocols. **B.** Bar chart presentation of flow cytometry analysis. Data represent mean ± SEM from 3 independent samples.
(TIF)

Table S1 Survival and symptoms observed in mice after infection with different *Cpn* doses.
(DOCX)

Table S2 Sequence and positivity of the primers on the OmpA gene encoding *Cpn* MOMP.
(DOCX)

Table S3 Statistical analysis of the effect of immunization with the peptides.
(DOCX)
