# Correction: Immunization of *Chlamydia pneumoniae* (*Cpn*)-Infected Apob^tm2Sgy^Ldlr^tm1Her^/J Mice with a Combined Peptide of *Cpn* Significantly Reduces Atherosclerotic Lesion Development

**DOI:** 10.1371/journal.pone.0099474

**Published:** 2014-05-30

**Authors:** The PLOS ONE Staff

In the Funding section, the grant from the Institute of Medical Microbiology and Immunobiology, University of Szeged, Hungary by the Hungarian Government is listed incorrectly. The correct grant number is: TÁMOP-4.2.2.A-11-1-KONV-2012-0035.
