# Supplementary figures and images for: Immunization of Chlamydia pneumoniae (Cpn)-Infected Apobtm2SgyLdlrtm1Her/J Mice with a Combined Peptide of Cpn Significantly Reduces Atherosclerotic Lesion Development
Source: PLoS One. 2013 Dec 13;8(12):e81056. doi: 10.1371/journal.pone.0081056 (PMC3862476; doi:10.1371/journal.pone.0081056)

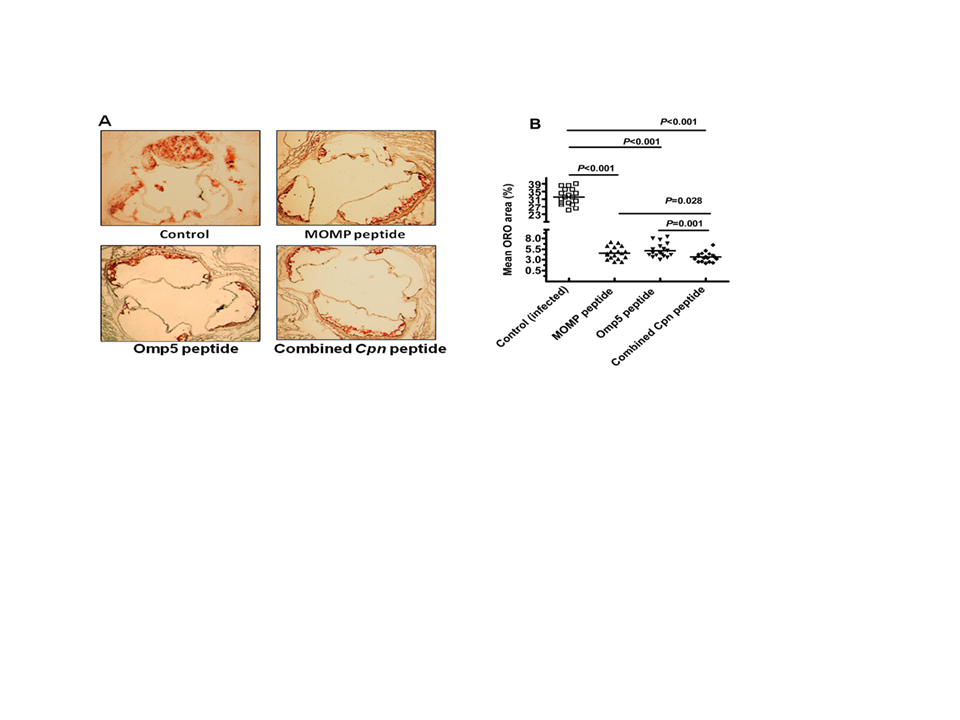

Supplement: Figure S1 — Detection and quantitation of lesion areas in the aorta of Ldlrtm1HerApobtm2SgyJ mice infected with Cpn bacteria and fed a high-fat diet after immunization with Cpn peptides vs controls infected with Cpn bacteria and fed a high-fat diet after immunization with KLH only. A. Representative photomicrographs of Oil Red O staining for lipids in cryosections of aortic root from immunized mice. Lipids are identified by red color. B. Quantification of ORO staining in the aortic root of Apobtm2SgyLdlrtm1Her/J mice. ORO stained area versus total area (%) at aortic roots (N = 18 sections, 3 sections per mouse). (TIF) [file pone.0081056.s001.tif]

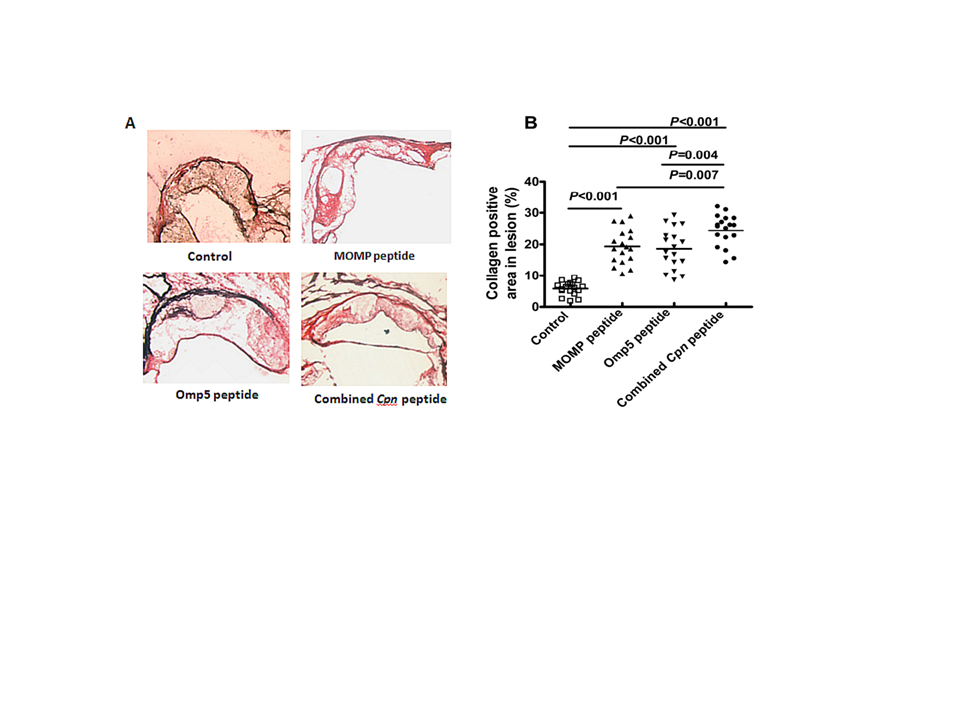

Supplement: Figure S2 — Detection and quantitation of collagen contents at lesion areas in the aorta of Ldlrtm1HerApobtm2SgyJ mice infected with Cpn bacteria and fed a high-fat diet after immunization with Cpn peptides vs controls infected with Cpn bacteria and fed a high-fat diet after immunization with KLH only. A, Representative photomicrographs and quantitative analysis of collagen (Sirius Red coloration under polarized light) in atherosclerotic aortas in individual mice B. Quantitation of collagen content at lesion area in the aorta of Apobtm2SgyLdlrtm1Her/J mice (N = 18 sections, 3 sections per mouse). (TIF) [file pone.0081056.s002.tif]

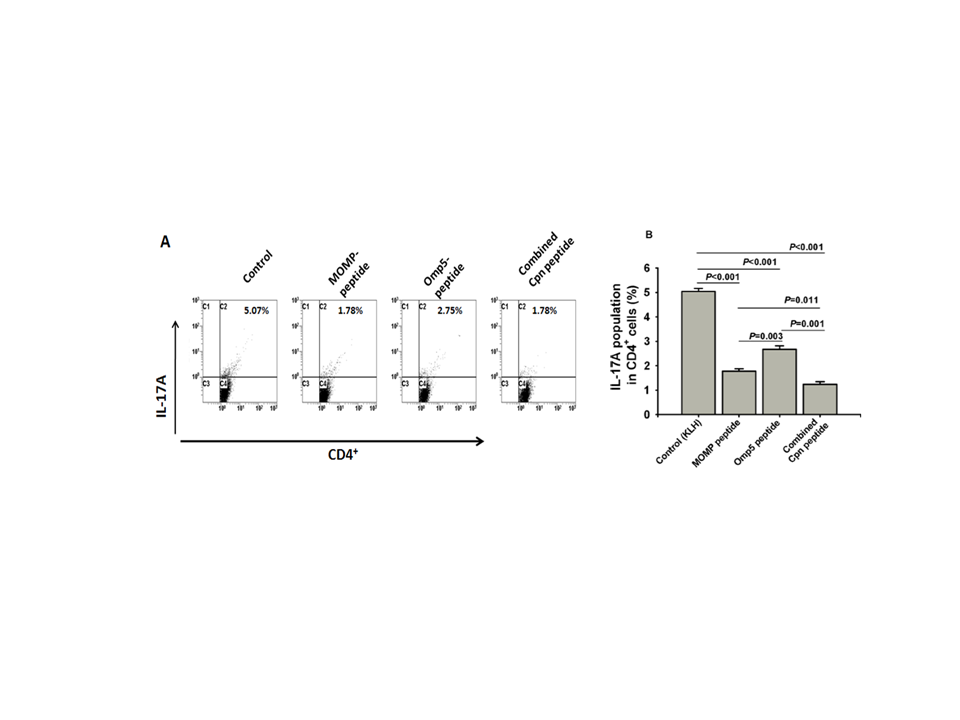

Supplement: Figure S3 — Assessment of IL-17A expression level in splenocytes from Ldlrtm1HerApobtm2SgyJ mice infected with Cpn bacteria and fed a high-fat diet after immunization with Cpn peptides vs controls infected with Cpn bacteria and fed a high-fat diet after immunization with KLH only. A. Representative flow cytometry plots for IL-17A expressing CD4+ population in spleen cells. Spleen cells from mice infected with Cpn bacteria and fed a high-fat diet after immunization with Cpn peptides and control mice infected with Cpn bacteria and fed a high-fat diet after immunization with KLH only were purified using a CD4+ purification kit (Miltenyi Biotec, Surrey, UK) according to manufacturer's protocols. B. Bar chart presentation of flow cytometry analysis. Data represent mean ± SEM from 3 independent samples. (TIF) [file pone.0081056.s003.tif]
