# Supplementary material for: Immunization of Chlamydia pneumoniae (Cpn)-Infected Apobtm2SgyLdlrtm1Her/J Mice with a Combined Peptide of Cpn Significantly Reduces Atherosclerotic Lesion Development
Source: PLoS One. 2013 Dec 13;8(12):e81056. doi: 10.1371/journal.pone.0081056 (PMC3862476; doi:10.1371/journal.pone.0081056)
Supplement: Table S1 — Survival and symptoms observed in mice after infection with different Cpn doses. (DOCX) [file pone.0081056.s004.docx]

**Table S1.**  Survival and symptoms observed in mice after infection with different *Cpn* doses

| **Dose used** | **No. of Mice used** | **Survival and disease signs** |
| --- | --- | --- |
| 4x10^6^ IFU | 6 | 3/6 were dead on day one, 3/6 showed severe signs of disease: ruffled fur, hunched back, severely reduced activity until day 6 post-infection |
| 2x10^6^ IFU* | 6 | slightly ruffled fur, moderately reduced activity until day 5 post-infection, all mice survived |
| 1x10^6^ IFU | 6 | no signs of sickness |

*We have chosen the dose which caused disease and was not a lethal dose.
