# Supplementary material for: Immunization of Chlamydia pneumoniae (Cpn)-Infected Apobtm2SgyLdlrtm1Her/J Mice with a Combined Peptide of Cpn Significantly Reduces Atherosclerotic Lesion Development
Source: PLoS One. 2013 Dec 13;8(12):e81056. doi: 10.1371/journal.pone.0081056 (PMC3862476; doi:10.1371/journal.pone.0081056)
Supplement: Table S2 — Sequence and positivity of the primers on the OmpA gene encoding Cpn MOMP. (DOCX) [file pone.0081056.s005.docx]

**Table S2.** Sequence and positivity of the primers on the OmpA gene encoding *Cpn* MOMP.

| **Primers** | **sequence** | **Positive** |
| --- | --- | --- |
| **external** | **333 base-pair products** |  |
| **Cpn1 (forward)** | **5’ TTA CAA GCC TTG CCT GTA GG 3’** | **61–80** |
| **Cpn2 (reverse)** | **5’GCG ATC CCA AAT GTT TAA GGC 3’** | **373–393** |
| **Internal** | **207 base-pair products** |  |
| **Cpn3 (forward)** | **5’ TTA TTA ATT GAT GGT ACA ATA 3’** | **100–120** |
| **Cpn4 (reverse)** | **5’ ATC TAC GGC AGT AGT ATA GTT 3’** | **286–306** |
