# Supplementary material for: Immunization of Chlamydia pneumoniae (Cpn)-Infected Apobtm2SgyLdlrtm1Her/J Mice with a Combined Peptide of Cpn Significantly Reduces Atherosclerotic Lesion Development
Source: PLoS One. 2013 Dec 13;8(12):e81056. doi: 10.1371/journal.pone.0081056 (PMC3862476; doi:10.1371/journal.pone.0081056)
Supplement: Table S3 — Statistical analysis of the effect of immunization with the peptides. (DOCX) [file pone.0081056.s006.docx]

**Table S3.** Statistical analysis of the effect of immunization with the peptides

|  | MOMP peptide  (A) | OMP5 peptide  (B) | Combined *Cpn* peptide  (C) | Statistical analysis |
| --- | --- | --- | --- | --- |
| Lesion occupied areas in aortas (%) | 19 | 18 | 14 | C vs A, *P*<0.05 |
| Lesion reduction in aortas (%) | 39.8 | 41.7 | 54.7 |  |
| Lesion occupied areas in descending aortas (%) | 5.8 | 7.8 | 3.4 | C vs B, *P*<0.01 |
| Lesion reduction in descending aortas (%) | 81.9 | 71.5 | 88.9 |  |
| ORO stained lipid area (%) | 4.6 | 5.2 | 3.6 | C vs A, *P*<0.01  C vs B, *P*<0.01 |
| Collagen content in lesions (%) | 19.36 | 18.59 | 24.39 | C vs A, *P*<0.01  C vs B, *P*<0.01 |
| Macrophage-occupied area in lesion (%) | 12.3 | 12.2 | 7.8 | C vs B, *P*<0.05 |
| Dendritic cell-occupied area (%) | 8.3 | 8.4 | 5.2 | C vs A, *P*<0.01  C vs B, *P*<0.01 |
| CD4^+^ T cell expressing Foxp3 (%) | 8.4 | 10 | 21.4 | C vs A, *P*<0.05  C vs B, *P*<0.05 |
| CD4^+^ T cell expressing IL-10 | 6 | 5.6 | 9 |  |
| TNF-α-occupied areas in lesions | 9.2 | 9 | 6 | C vs A, *P*<0.05 |
| IL-10 in plasma (pg/ml) | 30 | 26.3 | 42.3 | C vs A, *P*<0.05  C vs B, *P*<0.05 |
| TGF-β in plasma (pg/ml) | 24 | 25 | 42.6 | C vs A, *P*<0.01  C vs B, *P*<0.01 |
| TNF-α in plasma (pg/ml) | 201.7 | 198.3 | 177.3 | C vs A, *P*<0.05  C vs B, *P*<0.05 |
| IFN-γ in plasma (pg/ml) | 33 | 33 | 27 | C vs B, *P*<0.05 |
| IL-10 in supernatants of splenocytes (pg/ml) stimulated with 10 μg/ml ConA | 25.7 | 24.7 | 30 | C vs A, *P*<0.05  C vs B, *P*<0.05 |
| TGF-β in supernatants of splenocytes (pg/ml) stimulated with 10 μg/ml ConA | 33 | 33 | 43 | C vs A, *P*<0.05  C vs B, *P*<0.05 |
| TNF-α in supernatants of splenocytes (pg/ml) stimulated with 10 μg/ml ConA | 225 | 221 | 201 | C vs A, *P*<0.05  C vs B, *P*<0.05 |
| IFN-γ in supernatants of splenocytes (pg/ml) stimulated with 10 μg/ml ConA | 32 | 32 | 27 | C vs A, *P*<0.05  C vs B, *P*<0.05 |
| IL-17A expressing CD4^+^ T cells | 1.8 | 2.7 | 1.2 | C vs A, *P*<0.05  C vs B, *P*<0.01 |
